# Evaluation of Oxford Nanopore Technologies MinION Sequencer as a Novel Short Amplicon Metabarcoding Tool Using Arthropod Mock Sample and Irish Bat Diet Characterisation

**DOI:** 10.1002/ece3.71333

**Published:** 2025-05-04

**Authors:** James M. Nolan, Ilze Skujina, Gwenaëlle Hurpy, Andrew J. Tighe, Conor Whelan, Emma C. Teeling

**Affiliations:** ^1^ School of Biology and Environmental Science University College Dublin Dublin 4 Ireland; ^2^ School of Biological and Chemical Sciences University of Galway Galway Ireland; ^3^ Department of Biological Sciences Pwani University Kilifi Kenya

**Keywords:** amplicon sequencing, applied ecology, biomonitoring, diet analysis, environmental DNA, nanopore

## Abstract

Biodiversity monitoring using metabarcoding is now widely used as a routine environmental management tool. However, despite the rapid advancement of third‐generation high‐throughput sequencing platforms, there are limited studies assessing the most suitable tools and approaches for environmental metabarcoding studies. We tested the utility of Oxford Nanopore Technologies MinION sequencing for short‐read amplicon sequencing of mitochondrial *COI* mini‐barcodes from a known composition of arthropod species and compared its performance with more commonly used Illumina NovaSeq sequencing. The mock arthropod species assemblage allowed us to optimise a bioinformatic filtering pipeline to identify arthropod species using MinION long reads. Using this pipeline, we identified host species and diet composition by sequencing droppings collected from five individual Irish brown long‐eared bats ( *Plecotus auritus* ) roosts. We showed that MinION data provided a similar taxonomic assignment to *NovaSeq* but only if the reference species barcode database was accurate and comprehensive. The *P. auritus* diet inferred was as expected based on previous morphological and Illumina metabarcoding studies. We showed that less sequencing depth, but a higher number of biological samples were necessary for complete species composition detection by MinION. A relatively simple bioinformatic filtering tool such as NanoPipe could adequately retrieve both host species and diet composition. The biggest standing challenge was the reference database format transferability and comprehensiveness. This pipeline can be used to guide future metabarcoding studies using nanopore sequencing to minimise the cost and effort while optimising results.

## Graphical abstract

Oxford Nanopore Technologies MinION (ONT) Nanopore‐NanoPipe pipeline was compared to Illumina‐Obitools command‐line pipeline by using arthropod mock community input DNA for metabarcoding analysis. We demonstrated that a relatively simple ONT sequencing metabarcoding procedure followed by user‐friendly bioinformatics processing by graphical interface NanoPipe can be used to reliably and quickly characterise bat diet from faeces and thus has potential for other biodiversity monitoring programs.

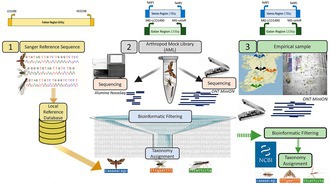

## 1 Introduction

Next generation sequencing technologies (NGST) have driven innovative ways to complement or possibly even replace traditional biodiversity assessment methods (Compson et al. 2020). NGST in combination with the generation of large public reference databases of common “DNA barcode” genes provides a fast, powerful, and more high‐throughput species identification method, using DNA retrieved from the environment (eDNA) such as water (Bracken et al. 2019; Sales et al. 2020; Schneider et al. 2016), sediment (Angeles et al. 2023; Mauffrey et al. 2021), soil (Ariza et al. 2023; Kirse et al. 2021), scat (Biffi et al. 2017; Galan et al. 2018; Jusino et al. 2019; Curran et al. 2022) and even air (Lynggaard et al. 2022; Métris and Métris 2023). The biggest advantage of eDNA metabarcoding arguably is its non‐reliance on the capture and physical examination of organisms and required morphological expertise of clade‐specific taxonomists, which provides a fast and often more accurate species assemblage measurement (Lejzerowicz et al. 2015; Fernández et al. 2019). It has transformed many biological fields, such as conservation management (Lopes et al. 2021; Sahu et al. 2022), ecological studies (Deiner et al. 2017; Tournayre et al. 2021), environmental biomonitoring (Andrés et al. 2023; Mauffrey et al. 2021; Gold et al. 2021), agriculture and aquaculture (Sow et al. 2020; Dully et al. 2021; Montauban et al. 2021; Ando et al. 2022), and even public health and food security (Charalampous et al. 2019; Shim et al. 2023; Watanabe et al. 2023). eDNA‐based biomonitoring is rapidly becoming an indispensable and routine tool for a wide range of stakeholders in industry (Imanian et al. 2022; Maiello et al. 2022), policy making (Hering et al. 2018; Mauffrey et al. 2021; Hestetun et al. 2021; Handley et al. 2023; Fernandez et al. 2022) and non‐governmental organisations (Lavin 2022; Ter Schure et al. 2021; Sahu et al. 2022). However, its widespread use is still limited by a lack of access to the expertise, laboratory and computational facilities, and budget required for the molecular data generation and bioinformatic analyses. These are currently the main barriers to reliable and efficient metabarcoding application for routine eDNA‐based biodiversity assessments.

Referred to as a third‐generation sequencing (TGS), Oxford Nanopore Technologies (ONT) is an alternative HTS technology for long‐read genomic studies (Jain et al. 2016; Lu et al. 2016). Potentially, it can also be used to conduct real‐time eDNA‐based biodiversity assessments due to its small instrument size and portability. Currently, the biggest disadvantage of ONT is a relatively high sequencing error rate of ~5% compared to other TGS methods (Amarasinghe et al. 2020; Santos et al. 2020). Recent improvements to basecalling and newer flow‐cell models have decreased sequencing error for short reads using ONT instruments, which has resulted in its successful application in environmental DNA barcoding studies (Chang et al. 2020; Van Der Reis et al. 2023). However, the three remaining bottlenecks of using ONT in metabarcoding analyses are: (1) limited accuracy comparison studies with the more established short‐read (e.g., Illumina) sequencing datasets, although see (Van Der Reis et al. 2023; Chang et al. 2020); (2) clear guidelines on how to process ONT sequencing datasets into relevant taxon tables bioinformatically; (3) user‐friendly and easy‐to‐access sequencing data analysis platforms suitable for ONT data formats. Recently emerging tools such as NanoPipe (Shabardina et al. 2019), which are designed specifically for ONT read genome assembly, offer promising avenues for long‐read metabarcoding processing; however, there are few validation studies of these tools based on reads generated from short amplicons. Overcoming these issues would enable the ONT platforms to be more commonly applied as potentially faster, more cost‐effective, and straightforward biodiversity assessment tools, particularly in applied field ecology.

To address these issues, we performed a case study using an artificial arthropod mock community and an empirical sample of Irish bat faeces collected in the field to test the accuracy, available metabarcoding software packages and parameters, and practicality of the ONT MinION sequencer to identify multiple prey species from bat droppings. As insectivores, bats are “keystone‐species” with an important role in ecosystem functioning and agricultural pest suppression. Furthermore, bats can be used as an indicator species of anthropogenic environmental impacts, such as the assessment of water quality and artificial lighting (Straka et al. 2020). Yet, as small, highly mobile and nocturnal species, their diets are difficult to study by direct observation or by morphological methods from faeces (Clare et al. 2011; Galan et al. 2018; Nolan et al. 2023). Elucidating the dietary components of a species is critical in order to fully understand trophic interactions within an ecosystem (Bregman et al. 2015; Ingala et al. 2018) and metabarcoding of bat faeces on Illumina platforms has proven to be a successful and efficient solution in numerous bat diet studies (Clare et al. 2011; Curran et al. 2022; Ingala et al. 2021; Mata et al. 2021). To the best of our knowledge, long‐read ONT technology has not been tested on bat faeces for diet metabarcoding.

Here we performed the first empirical ONT MinION metabarcoding study on Irish bat faeces. We directly compared Illumina NovaSeq and ONT MinION sequencing of the same arthropod mock library (AML) on samples collected in the field. We generated an optimal pipeline for analyses and provide practical advice for using ONT for metabarcoding sequencing of eDNA.

## 2 Methods and Materials

### 2.1 Experimental Strategy

**FIGURE 1:**
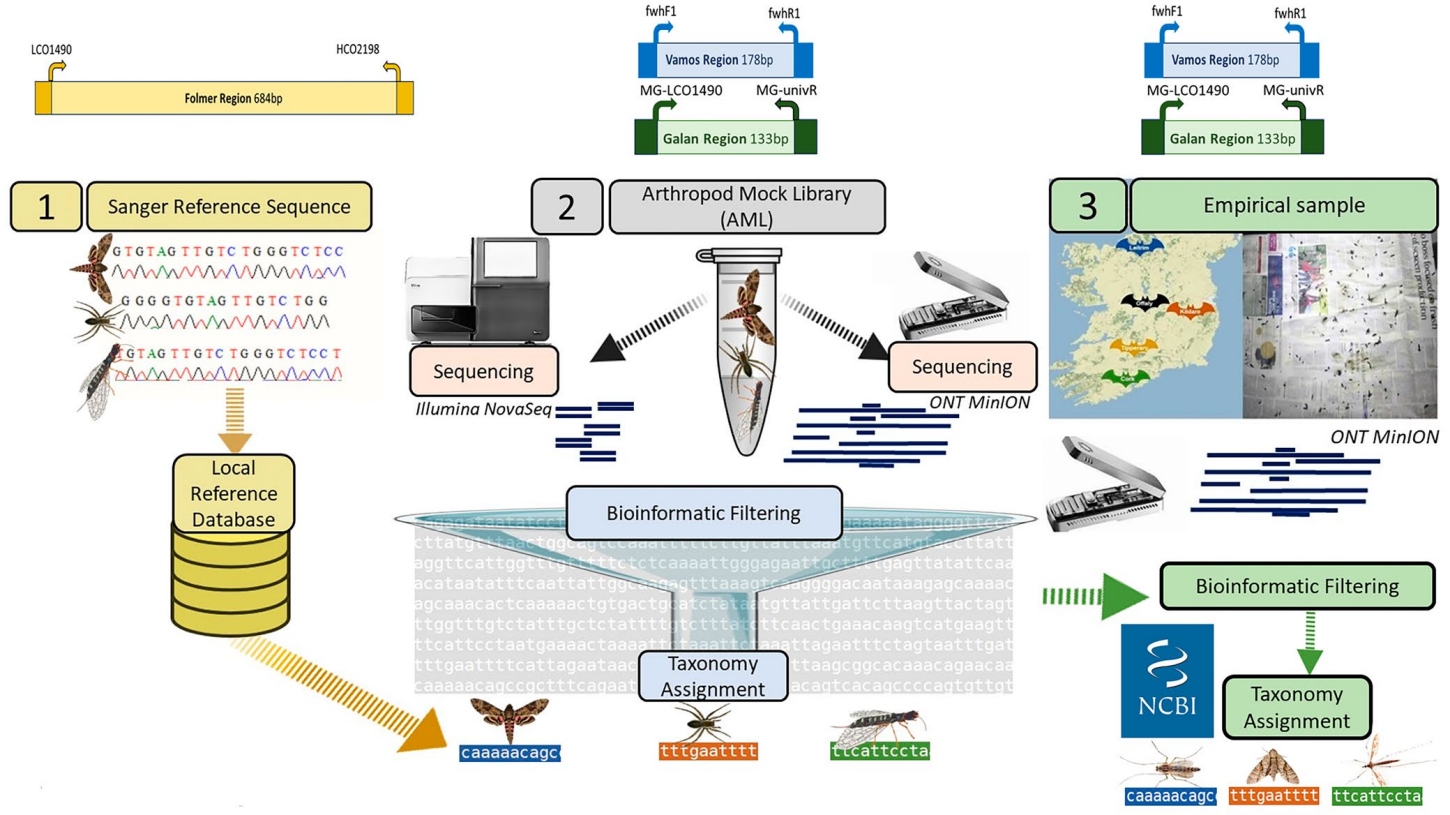
Overview of the AML experimental design for *Illumina* and *ONT* instrument comparison using known arthropod DNA pool and an empirical sample of Irish bat faecal pellets.

To assess the suitability, accuracy, and practicality of the ONT MinION sequencer for taxonomic identification of environmental samples, we firstly used an AML with known exact reference sequences obtained by Sanger sequencing of each individual animal before pooling. The same arthropod pool was split and sequenced on both ONT and Illumina platforms. This allowed us to directly assess the reliability of ONT MinION performance compared to more established Illumina HTS and to evaluate the effect of bioinformatic parameter choice at each step to design an optimised workflow. This optimised workflow was then used for ONT MinION sequencing of a real‐life sample of Irish bat  droppings (Figure 1).

### 2.2 Arthropod Mock Library (AML)

**FIGURE 2:**
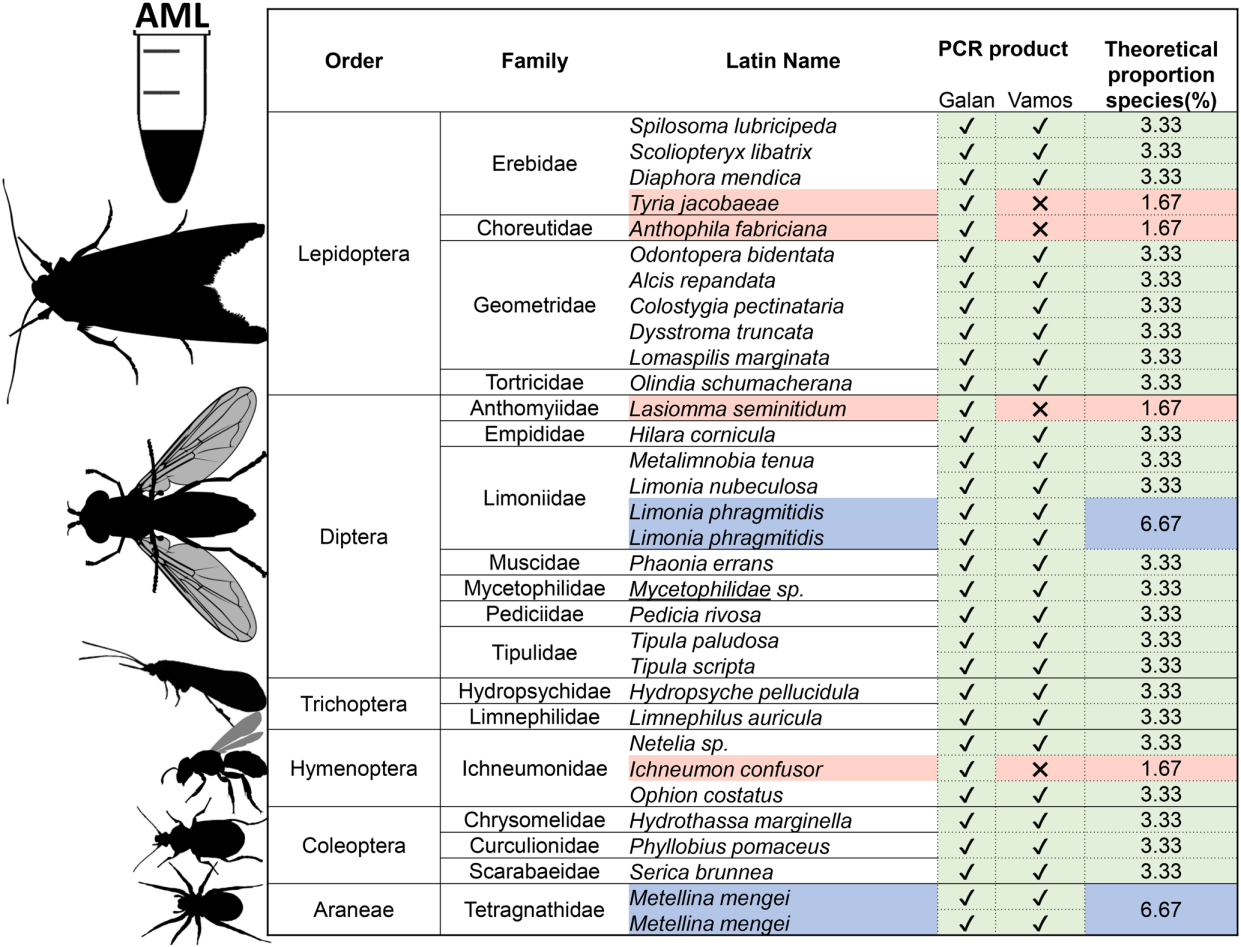
Composition of the artificial Arthropod Mock Library consisting of Galan and Vamos PCR amplicons for each of the listed individuals apart from the four highlighted in red lacking Vamos amplicon. The species highlighted in blue were represented by two different individuals.

The arthropods were caught in the direct vicinity of the bat roosts in Ireland in Glengarriff (Co. Cork), Letterfrack (Co. Galway) and Kilmore (Co. Cavan) as part of an ongoing Irish bat diet study (Hurpy, unpublished data). All arthropod collections were made in full compliance with local, national, ethical, and regulatory principles, and required licences for light trap and Lepidoptera collection were obtained (No. C 161/2021 and No. C66/2022, respectively). The arthropods were collected using sweep net, beat tray, malaise trap, and light trap, and then stored in 95% ethanol. A total of 32 arthropods were identified to species/family level using either morphological identification (Waring and Townsend 2017) and/or molecular barcoding of the Folmer metabarcoding region (Folmer et al. 1994). Individual arthropods were chosen from available species in the laboratory (Table S1). These represented 30 species, 28 genera, 18 families and six orders (Figure 2), all of which are potential prey of Irish bats. The number of species was typical of that used in other metabarcoding mock assemblages (Iwaszkiewicz‐Eggebrecht et al. 2023).

### 2.3 DNA Extraction and Amplification

#### 2.3.1 Arthropod DNA Extraction

Arthropod DNA was extracted from a small piece (~2–3 mm) of leg tissue using a standard chelating resin—chelex (InstaGene Matrix, Bio‐Rad) technique (Singer‐Sam 1989; Walsh et al. 1991). Briefly, tissue was placed in a 1.5 mL microcentrifuge tube with 300 μL of 10% chelex containing 0.2 mg/mL proteinase K (Merck Life Science Limited). The leg tissues were mechanically sheared using sterile scissors, vortexed, and incubated for 1 h at 56°C and 500 rpm, and then vortexed again and boiled at 99°C for 15 min to inactivate the proteinase K. The sample was then centrifuged twice at 14,000 rpm for 1 min, first retaining 150 μL, and then 100 μL of final supernatant volume containing the DNA.

#### 2.3.2 Empirical Case Study Sample Collection and DNA Extraction

A total of five bat roosts located in County Tipperary, Offaly, Kildare, Cork, and Leitrim were selected for this study via Bats & Bugs (https://batsandbugs.ie/) citizen science project. Fresh faecal pellets were collected by placing newspaper on the floor of the bat roosts for 2 days to ensure the adequate collection of fresh droppings. The newspaper helped prevent humidity and consequently avoided DNA degradation (Puechmaille and Petit 2007; Puechmaille et al. 2007). The droppings were collected using sterile forceps and placed in Eppendorf tubes. Five individual droppings per roost were homogenised using a mortar and pestle. DNA was extracted from a 0.2‐g subsample of the homogenised droppings using the NucleoSpin 96 Plant II Kit (Macherey‐Nagel) following the optimised manufacturer's protocol detailed by (Zarzoso‐Lacoste et al. 2018). To minimise cross‐contamination from other eDNA studies, all faecal DNA extractions were performed in a designated pre‐PCR laboratory within a sterile laminar flow hood. The mortar and pestle were cleaned using 20% bleach and 70% ethanol between each use to minimise cross‐contamination. A blank sample containing no guano lysate was added and processed exactly the same way as the other faecal samples. The blank sample lysate was treated in the mortar and pestle and then subjected to the same laboratory procedures. This DNA extraction negative control and PCR negative control (water) ensured no cross‐contamination had occurred and allowed us to evaluate the potential for spurious amplification and sequencing that can occur in all metabarcoding studies.

#### 2.3.3 Sanger Reference Sequence Generation for AML

Extracted arthropod DNA was amplified using primers from Folmer et al. (1994) spanning the entire common metabarcoding DNA section of the mitochondrial *cytochrome oxidase I* (*COI*) gene (Table 1). All PCRs were carried out in a total volume of 25 μL and included the following reagents: 16.3 μL of PCR grade water, 1.5 μL of MgCl_2_ at 25 μM, 2.5 μL of 10× *Platinum* PCR buffer, 0.5 μL of DNTPs at 10 μM, 0.83 μL of each primer both at 10 μM, 0.83 μL Bovine Serum Albumin (BSA), 0.18 μL 5 U/mL *Platinum Taq DNA Polymerase* (Invitrogen) and 1.5 μL of DNA template. The thermocycle profile used was: 95°C for 5 min followed by 6 cycles of 94°C for 1 min, 47°C for 80s and 72°C for 70s, then 36 cycles of 94°C for 1 min, 53°C for 80s and 72°C for 70s and a final extension of 72°C for 5 min. A negative PCR control containing sterile water instead of target DNA was used to exclude cross‐contamination. The PCR amplicons were cleaned up with 0.9 vol *AMPure XP* beads (Beckman Coulter) and sent for bidirectional Sanger sequencing (Macrogen, https://www.macrogen‐europe.com) with each of the LCO1490 and HCO2198 primers.

**TABLE 1:** Primer pairs used throughout this study.

Primer pair name	Name	Forward primer sequence	Name	Reverse primer sequence	PCR Amplicon length	Purpose	Source
Folmer	LCO1490	5′‐GGTCAACAAATCATAAAGATATTGG‐3′	HCO2198	5′‐TAAACTTCAGGGTGACCAAAAAATCA‐3′	658 bp	AML highly accurate reference sequence from individual arthropods	Folmer et al. (1994)
Vamos	fwhF1	5′‐YTCHACWAAYCAYAARGAYATYGG‐3′	fwhR1	5′‐ARTCARTTWCCRAAHCCHCC‐3′	178 bp	To amplify and sequenced pooled AML for Illumina and ONT libraries; To amplify and ONT sequence bat faecal droppings for empirical sample	Vamos et al. (2017)
Galan	MG‐LCO1490	5′‐ATTCHACDAAYCAYAARGAYATYGG‐3′	MG‐univR	5′‐ACYATRAARAARATYATDAYRAADGCRTG‐3′	133 bp	To amplify and sequenced pooled AML for Illumina and ONT libraries; To amplify and ONT sequence bat faecal droppings for empirical sample	Gillet et al. (2015), Galan et al. (2018)

#### 2.3.4 Arthropod Short Metabarcoding Region Amplification

Two sets of short metabarcoding amplicon primers – “Galan” and “Vamos” (Galan et al. 2018; Vamos et al. 2017) ‐ designed specifically for arthropod studies were used to amplify each individual for AML pool and for the real‐life case‐study library creation from the faecal pellets (Table 1). Both Galan and Vamos primer sets amplify a broad range of arthropod and host. The amplified length is 133 and 178 bp for Galan and Vamos, respectively, and both forward primers are located at the same 5′ position of the LCO1490 within the Folmer region (Tournayre et al. 2020), while the Vamos forward primer contains more degenerative nucleotides (Table 1). The PCR reactions for AML ONT and Illumina sequencing library pool using Vamos and Galan primer sets were carried out separately for each individual arthropod used in the mock library as described in *2.3.3*. The thermocycle profile was as follows: 95°C for 5 min, followed by 40 cycles of 95°C for 30 s, 47°C for 45 s, and 72°C for 1 min, and a final extension of 72°C for 5 min. A negative control (containing 1.5 μL of PCR grade water instead of the template DNA) and a positive control of genomic DNA from the greater mouse‐eared bat, *Myotis myotis* , was included in each PCR amplification batch. The PCR products were cleaned using 1 vol *AMPure XP* beads (Beckman Coulter).

#### 2.3.5 AML Pooling and Estimation of Theoretical Species Composition

The purified metabarcoding PCR product DNA concentration was calculated using the *Qubit dsDNA BR* fluorometer assay (Invitrogen), then amplicons were pooled in a single tube in equimolar concentrations of 5.15 ng/μl (Table S2). The AML sample size and integrity were assessed by agarose gel electrophoresis. We chose to retain and pool all Galan and Vamos amplicons, although four species only amplified with Galan but not with Vamos primers (i.e., *Tyria jacobaeae, Anthophila fabriciana, Lasiomma seminitidum, Ichneumon confusor*) despite repeating the PCR. Expected (theoretical) proportions for each species were calculated based on the total number of PCR amplicons per species in the equimolar library pool of the mock community with the highest input proportion (6.67%) for *Limonia phragmitidis* and *Metellina mengei* that were represented by two individuals per species (Figure 2), lowest—the above‐mentioned *T. jacobaeae, A. fabriciana, L. seminitidum, I. confusor* (1.67%) and 3.33% for all the rest of the species.

Using pooled PCR amplicons, rather than pooled bulk DNA as in previous studies e.g. Galan et al. (2018), allowed us to exclude PCR amplification bias and errors (Braukmann et al. 2019). The library was then split in half for *ONT* and *Illumina* sequencing (described below in 2.5 and 2.6).

### 2.4 Sanger Reference Sequences (“Highly Specific” Database)

Geneious v.2022.2 was used to assemble LCO1490 and HCO2198 Sanger sequences into a single consensus sequence for each of the individual arthropods used in the AML. Sequence traces were checked, and only the bases with unambiguous peaks were used. Sequence species identity was checked by using the NCBI BLAST web interface (Altschul et al. 1990; Johnson et al. 2008) in September 2023 (Table S1). The sequences were then stored in FASTA format and transformed into a local BLAST database format using the *makeblastdb* command within *blast v.2.12.0*, further referred to as “highly specific” reference database (HS). The *de novo* reference sequences from the Irish arthropods generated during this study have been deposited in the NCBI GenBank under accession numbers (PP759737‐ PP759766, PQ187542, PQ187543).

### 2.5 AML *Illumina NovaSeq* Sequencing and Bioinformatic Workflow

A total amount of 0.26 μg AML DNA (56 μL) was sent for sequencing to *Novogene UK* (https://www.novogene.com/eu‐en/) using the *Illumina NovaSeq 6000* PE150. The AML was prepared for sequencing at the *Novogene* laboratory using PCR‐free library preparation (*Novogene Plant and Animal PCR product Whole Genome Sequencing (WOBI)* protocol), which essentially entailed an end‐repair, A‐tailing, and further ligation with Illumina adapters. We requested 2Gb of data (equivalent to 6.6 M PE150 reads), which was the minimum amount for this sequencing service. Since the *NovaSeq* reads were produced by a commercial sequencing company (*Novogene*) and via a paired‐end (PE) sequencing strategy, the quality was assessed after merging the 5′ and 3′ reads into a single aligned read with a minimum of 40 alignment score (*n* = 6,169,038) using *FastQC v.0.11.9* (*Babraham Bioinformatics*).

Filtering steps and construction of the molecular operational taxonomic units (MOTUs) were conducted using *OBITools v1.01.22* (Boyer et al. 2016) pipeline and custom R scripts by Wangensteen (https://github.com/metabarpark/R_scripts_metabarpark) [see Figure 4]. Firstly, the paired‐end reads were aligned with *illuminapairedend*, only retaining the reads with an alignment score higher than 40. Removal of primer sequences was done using *ngsfilter* (*OBITools v1.01.22*). Rare sequence variants and artefacts that likely originated due to PCR or sequencing errors were discarded by first dereplicating the reads using *obiuniq* and then filtering with *obigrep* (*OBITools v1.01.22*). Only reads with the expected barcode region length between 133 and 178 bp and without any ambiguous nucleotides (N) were retained. We tested four different counts of identical reads during the dereplication step, only retaining sequences represented by more than 3, 5, 10, or 15 reads. Chimeric sequences were searched using the *uchime_denovo* function (Edgar et al. 2011) in the *VSEARCH* v2.17 package (Rognes et al. 2016), but none were detected. To cluster the individual unique sequences into MOTUs, *sumaclust v1.0.36* (Mercier et al. 2013) was tested with commonly used 98%, 97%, and 95% identity thresholds (Alberdi et al. 2018). MOTUs with an abundance of only one sequence variant (i.e., singletons) were removed with *obiclean* (*OBITools v1.01.22*). FASTA files containing the final clean MOTUs were tabulated using *obitab* (*OBITools v1.01.22*) and converted to *csv* tables by a custom script (Owen S. Wangensteen available https://github.com/metabarpark/R_scripts_metabarpark).

### 2.6 ONT MinION Sequencing and Bioinformatic Workflow

#### 2.6.1 ONT MinION Library Preparation

The AML and case‐study faecal pellet MinION libraries were prepared and sequenced using ONT SPOT ON FLO‐MIN106D (R9.4.1) and ONT SQK‐LSK109 Ligation Sequencing chemistry according to the manufacturer's protocol (“*Ligation sequencing gDNA (SQK‐LSK109)*” available at ONT *Community*). A dedicated single flow cell (FAQ90138) was used for the AML. The library preparation entailed DNA end‐repair and dA‐tailing using the *NEBNext End Repair/dA*‐*tailing* module, followed by the ligation of the ONT sequencing adapters (supplied in the kit) onto the prepared ends as per manufacturer's instructions.

The five roost faecal amplicon pools were first end‐repaired using *NEBNext End Repair/dA*‐*tailing* module and multiplexed using roost‐specific barcodes onto a different flow cell (FLO‐MIN106D) using *ONT EXP‐NBD196* barcoding kit following the *ONT* “*Ligation sequencing amplicons ‐ native barcoding (SQK‐LSK109 with EXP‐NBD196*)” as per manufacturer's instructions to be sequenced parallel to multiplexed samples from other projects.

The sequencing runs were carried out using *MinKNOW Core v3.6.5* (ONT) until all the pores were exhausted (24–48 h). Basecalling was performed using Guppy v3.2.10 (ONT) in high accuracy mode. *Porechop* v0.2.47 (https://github.com/rrwick/Porechop) was used to remove the adaptor sequences from the raw reads. To evaluate the quality and read length distribution of the raw sequencing data, we used the quality checking software *FastQC* v0.11.9 (*Babraham Bioinformatics*). Only the reads ranging between 110 and 220 bp were retained using *NanoFilt* 2.8.0 (De Coster et al. 2018; https://github.com/wdecoster/nanofilt), which is the expected size range for Galan and Vamos amplicons allowing a 20 bp window at each end.

#### 2.6.2 ONT MinION AML Bioinformatic Processing

*NanoFilt* was also used to filter the reads at three conventional quality levels for *ONT*: “high” (Q > 12), “medium” (Q > 10) and “low” (Q > 8) (Laver et al. 2015; Delahaye and Nicolas 2021). The three Q subsets were checked for chimeric reads using *VSEARCH* 2.17 (Rognes et al. 2016) *–uchime_denovo* function (Figure S1). *VSEARCH* was also used for dereplication (−*‐derep_fulllength*) and construction of the molecular operational taxonomic units (MOTUs) by unsupervised clustering method (*−‐cluster_fast* function). It was also compared to a supervised clustering method using *NanoPipe* (Shabardina et al. 2019).

**FIGURE 3:**
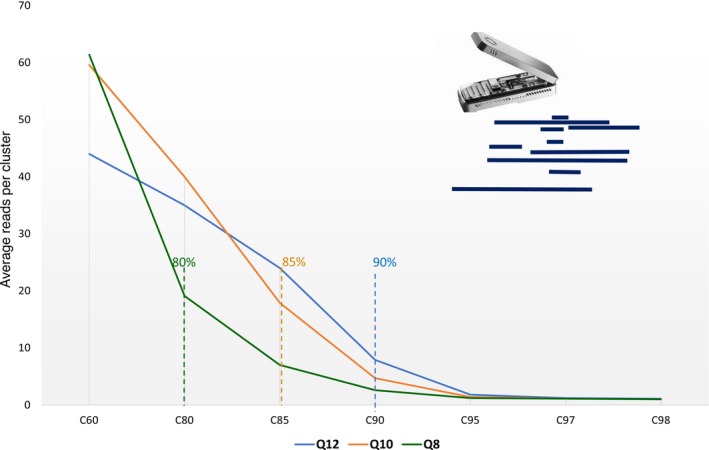
Number of average reads per cluster obtained at different values of *vsearch* clustering depending on the read quality: Read set with > Q12 (blue), > Q10 (yellow) and > Q8(green). The dotted lines of the respective colour show the point after which average number of reads start to plateau, representing a saturation point past which oversplitting will occur.

Due to the nature of nanopore sequencing and the wide read length distribution resulting from two different primer sets (Figures S2, S3; Table S3), the conventional unsupervised short amplicon read MOTU clustering approaches and thresholds are not suitable for nanopore data (Baloğlu et al. 2021). Therefore, for a reference‐free clustering, we first assessed a broad range of clustering thresholds of 60%, 80%, 85%, 90%, 95%, 97% and 98%. The optimal clustering threshold choice was somewhat subjective but was guided by the slope and absolute decrease of the average read number per cluster to construct a reasonable amount of MOTUs with sufficient depth (Bonin et al. 2023) [see Figure 3; Table S4].

Further, we assessed the effect of three different levels of read abundance per cluster supporting the cluster centres with a minimum of either 10, 50 and 100 reads using *VSEARCH ‐‐sortbysize ‐‐minsize* options. Cluster centres are the consensus sequences computed from a multi‐alignment of sequences that closely correspond to the true haplotype sequence. Finally, we also applied these clustering and abundance parameters on the three different sequence Phred score read quality sets “high” (Q > 12), “medium” (Q > 10) and “low” (Q > 8).

#### 2.6.3 Empirical Dataset Bioinformatics Processing

**FIGURE 4:**
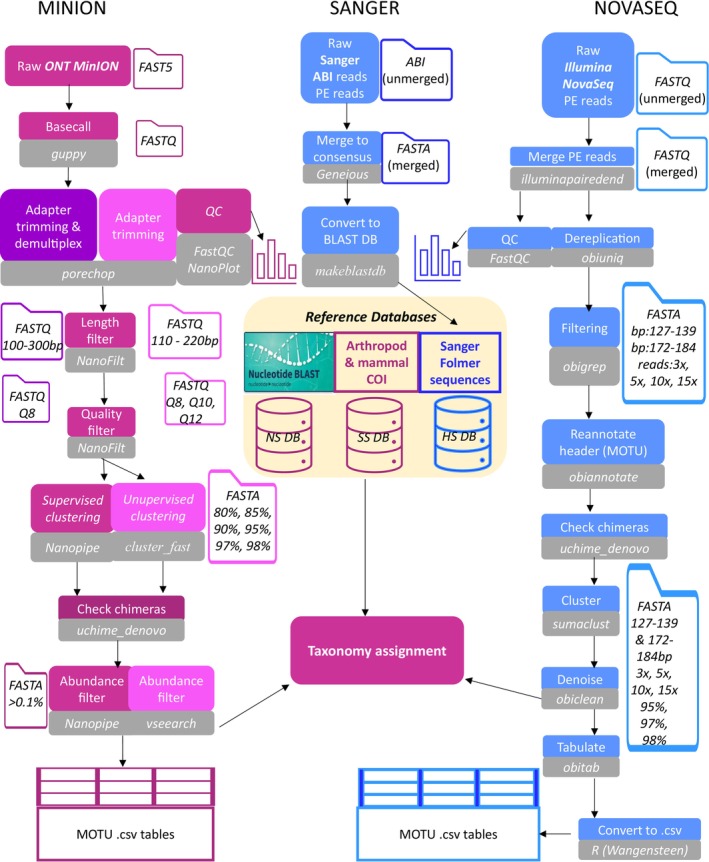
Overview of all main bioinformatics steps for Sanger, *ONT MinION*, *Illumina NovaSeq* data analysis. Each step is shown in a coloured box and grey boxes show the tool or specific command used. Blue boxes show the steps only applied for AML data; deep purple—only applied for the CS data; dark pink—show *ONT* analysis steps common for both AML and CS and light pink boxes show steps for *ONT* data applied only for AML data.

ONT MinION reads from the five roost samples were then filtered using the most optimal parameters as established from the AML analysis (Phred score > Q8) and length (100 bp—300 bp) using *NanoFilt* 2.8.0 (Figure 4). Clustering and MOTU construction was carried out using a reference‐guided approach on the *NanoPipe* webserver. A manually curated arthropod COI reference database was used containing all Irish and UK bat and all Irish mammal “Folmer” COI sequences retrieved from GenBank as of October 2023, as well as all arthropod “Folmer” Sanger sequences generated in this study (see Sections 2.3.3 and 2.4). The consensus clusters were further filtered based on read abundance, and only the clusters with a minimum of 0.03% of total clustered reads were retained as MOTUs used for the diet study based on our findings from the mock community reference tests (see Section 3.2). This was to ensure the avoidance of false positives in the final diet. The MOTUs were then taxonomically assigned using the *NCBI GenBank BLAST* web interface (Johnson et al. 2008) October 2023. A minimum of 97% identity and 90% query cover was required for a MOTU to be considered a representative of a true species. If a MOTU consensus sequence had more than one possible species of origin at the required parameters, it was referred to the BOLD database to determine the species of origin (Ratnasingham and Hebert 2007). Species that occurred in the extraction blank were also removed from the final diet analysis. All statistical analyses were carried out in *R v4.2.2* (*R Core Team,* *2022*) using packages *ggplot2* (Wickham et al. 2016), *vegan* (Oksanen et al. 2022), *plotly* (Sievert 2020), *ggpubr* (Kassambara 2023) and MASS (Venables et al. 2002).

### 2.7 AML Taxonomic Assignment

We analysed the effects of the three main factors that affect the accuracy of taxonomic assignment specific to each sequencing platform (Alberdi et al. 2018): (1) quality of the basecalling of sequence reads; (2) bioinformatic filtering parameter choice, which impacts the similarity between MOTUs; (3) the specificity and completeness of the reference database. To test the impact of the first two factors, we applied an unsupervised MOTU construction approach and assigned each MOTU to the known input species using a highly accurate and specific local Sanger reference sequence for each AML individual, i.e., “highly specific” reference database (HS). This allowed us to determine the filtering parameters that provided the taxonomic retrieval with the closest match to the expected read proportions for each species based on PCR product input for each AML arthropod. However, in real‐life scenarios, a complete and highly accurate database is not available. Thus, to gain insight into the outcome of “non‐specific” (NS) databases such as the entire *NCBI GenBank* database (accessed March 2023) and “semi‐specific” (SS), i.e., a local reference database consisting only of arthropod COI sequences (downloaded in March 2023), we used the MOTU set that provided the closest proportions to the input species from ONT and Illumina after the HS assignment.

The HS and SS assignment of the AML MOTUs was performed by the local *blastn* function in *blast* v.2.12, with 90% identity and 90% coverage for the *NovaSeq* dataset and 90% identity and 70% coverage for the ONT dataset against the Sanger sequence reference database. For the NS assignment, we used the *blastn ‐remote* option with 90% coverage and 97% identity for the Illumina and ONT. Finally, we also compared these results to a supervised MOTU construction approach implemented in the *NanoPipe* pipeline (Shabardina et al. 2019).

### 2.8 AML Statistical Analysis

In order to compare the performance of the two sequencing instruments and determine optimal bioinformatics parameters for taxonomic assignment, we followed (Nygaard et al. 2020). We used Spearman rank correlations to evaluate the mock sample closeness to the expected composition of the sequencing data produced by Illumina and ONT with each of the different filtering parameter combinations. The expected proportions were calculated based on the number of PCR products per taxonomic unit within the AML (Figure 2; Table S6). We considered the correlation strong if Spearman's Rho was 0.8–1, moderate for 0.5–0.8, weak for 0.2–0.5 and negligible if Rho was 0.0–0.2, and significant if *p* < 0.005. All statistical analyses and heatmap plots were generated in the *R* v4.2.2 (R Core Team, 2022) environment. To visualise species detected across sites and sequencing platforms, the heatmaps were prepared using the package *complexheatmap* v2.6.2 (Gu 2022). Evaluation of Illumina and ONT using the HS reference database was performed at the species level. The comparison of SS and NS reference databases used for taxonomic assignment was performed at the family level.

## 3 Results

### 3.1 AML Sequencing Results

Sanger sequencing of the Irish arthropods generated 32 *de novo* COI barcode sequences ranging from 611 to 688 bp. One of these barcode sequences was from a specimen currently only identified to genus level (*Netelia* sp.) based on morphology. It is a representative of an understudied group, as the closest match within *NCBI GenBank* had only 92.73% sequence identity (Table S1).

The Novogene AML run delivered 0.95Gb of sequence data within 14 days of sample send‐off. The total released 7,338,142 paired reads (14,676,284 total) were sequencing adapter trimmed and filtered to remove all the reads that contained ambiguous nucleotides and reads below average base count Q30 (Phred value > 30).

The AML ONT MinION ran for 21 h 20 m and resulted in 0.66Gb of data and approximately 1.9 million reads in FAST5 output. After basecalling, 1,866,307 total raw reads were generated.

The quality check showed that *Illumina NovaSeq* provided a very high‐quality sequencing data with the mean read quality between Q49–59, which was consistently above the conventional *Illumina* high‐quality cut‐off value of Q30 (inferred base call accuracy 99.9%) (Figure S4). The mean read quality Phred score for the *ONT MinION* data was Q8.5 (Figure S1; Table S3), which is above the minimum recommended threshold for *ONT* data output (Delahaye and Nicolas 2021).

### 3.2 AML Bioinformatic Analysis Results

#### 3.2.1 Filtering Parameter Choice

**FIGURE 5:**
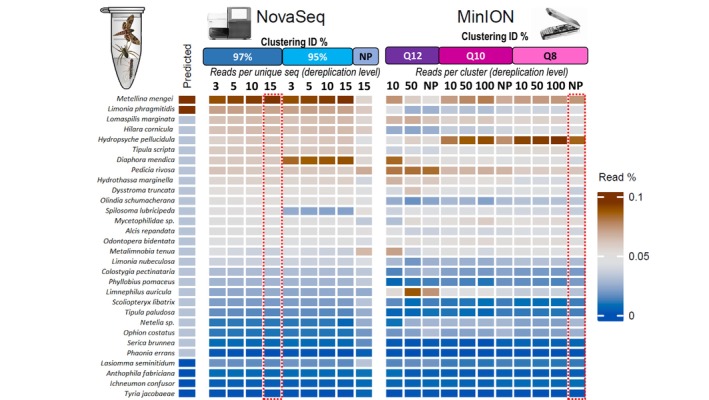
Expected versus observed AML compositions from *Illumina NovaSeq* and *ONT MinION* read output with various bioinformatic filtering parameters. Read number belonging to each species are depicted as percentage of total identified reads. Highlighted in red dashed lines are the most optimal pipeline parameter combination for each dataset. For *Illumina* data read dereplication was done at 3, 5, 10 and 15 read level and at 97%, 95% and 98% (not shown in the figure) cluster identity. For *MinION*—reads were filtered at Q12, Q10 and Q8 Phred score level and clustered based on 89%, 88% and 85% identity, respectively. These clusters were then further filtered based on minimum 10, 50 and 100 reads per cluster level (apart from Q12 reads, only 10 and 50 reads per cluster level was kept as at 50 some species were no longer detected). NP shows the results from guided clustering and taxonomic assignment using *NanoPipe* pipeline. Predicted proportions are calculated based on PCR product input. Spearman rank correlation strength and *P*‐values available in Table S6.

For the Illumina NovaSeq AML dataset, we ran the pipeline with the combinations of “conventional” unsupervised (reference‐free) 95%, 97%, 98% sequence clustering identity thresholds and dereplication levels of 3, 5, 10, and 15 identical reads per unique sequence (Figure 5; Tables S5 and S6). We also tested the effect of reference‐guided, i.e., supervised, filtering and clustering approach (using HS reference database) via the NanopPipe webserver. The closest taxonomic composition to the expected proportions was the same at the two highest clustering thresholds of 97% and 98% (Spearman's rank Rho = 0.62, *p* < 0.001; Table S6) and increased with the dereplication coverage level across all clustering thresholds, i.e., highest at 15 identical read support (Figure 5). Both approaches produced 185 final MOTUs and 1730 unique reads. The highest (15 identical reads) dereplication level also ensured the minimum number of unidentified reads (1.02%) at all clustering thresholds compared to up to 9.69% when only 3 identical reads were used for dereplication (Tables S5 and S6). Interestingly, the Rho dropped to 0.57 (*p* < 0.001) when a supervised (HS reference‐guided) NanoPipe clustering approach was used, even though the target file consisted of the exact individual Sanger sequences (Table S6). The two most falsely under‐represented species were *Phaonia errans* and *Serica brunnea* with only 0.08% and 0.61% of the total reads, respectively, instead of 3.33% as predicted based on the expected relative input reads. *Lomaspilis marginata*, *Hilara cornicula, Hydropsyche pellucidula*, and *Tipula scripta* were the most falsely over‐represented taxa (by ~70%) based on expected input.

The most optimal clustering level (89%, 88%, 85%) corresponding to reference‐free *ONT MinION* Phred Q12, Q10, or Q8 filtered reads was determined at a level where average reads per cluster did not considerably increase with the decrease of the sequence identity for the cluster threshold as described in 2.5 (Figure 3; Table S4). We further tested the outputs of these clustering and quality levels based on read depth: only keeping the cluster centres supported by over 10, 50, or 100 reads. With the unsupervised reference‐free clustering approach, the closest match to the expected taxonomic assemblage was with the Q8, 10 and 50 reads per cluster centre data sets (80% clustering threshold), but it was considered weak and insignificant (Spearman's rank Rho = 0.33, *p* = 0.07) and the amount of reads that were not assigned to any taxa was relatively high (34.99%, 38.41%, respectively). Spearman's rank correlation improved to medium if these unmapped reads were not considered (Spearman's rank Rho = 0.53, *p* < 0.005). The number of MOTUs produced this way (*n* = 7633) and unique reads (*n* = 968,913) was also high, which is bioinformatically taxing for further taxonomy assignment. Using HS reference‐guided consensus MOTUs via *NanoPipe* platform, considerably improved the read mapping to 92.3%, which assigned to 30 MOTUs correctly representing the 30 input species. The species composition of the expected proportions increased from weak to moderate Spearman's correlation of Rho 0.57 (*p* = 0.001, not considering unmapped reads) for the Q8 dataset (Table S6). Thus, contrary to *NovaSeq* reads, using a high specificity reference‐guided approach for MOTU construction on the *ONT MinION* dataset significantly improved accurate taxon retrieval. Similarly, for the *NovaSeq* dataset, the most falsely under‐represented species were *P. errans* and *S. brunnea* (3.61% and 18%, respectively, reads retrieved based on the expected relative input). *H. pellucidula*, *L. marginata* , *Pedicia rivosa* and *Mycetophilidae* sp. were among the most falsely over‐represented taxa (by 155% based on expected input) after *MinION* metabarcoding.

#### 3.2.2 Reference Database Choice

**FIGURE 6:**
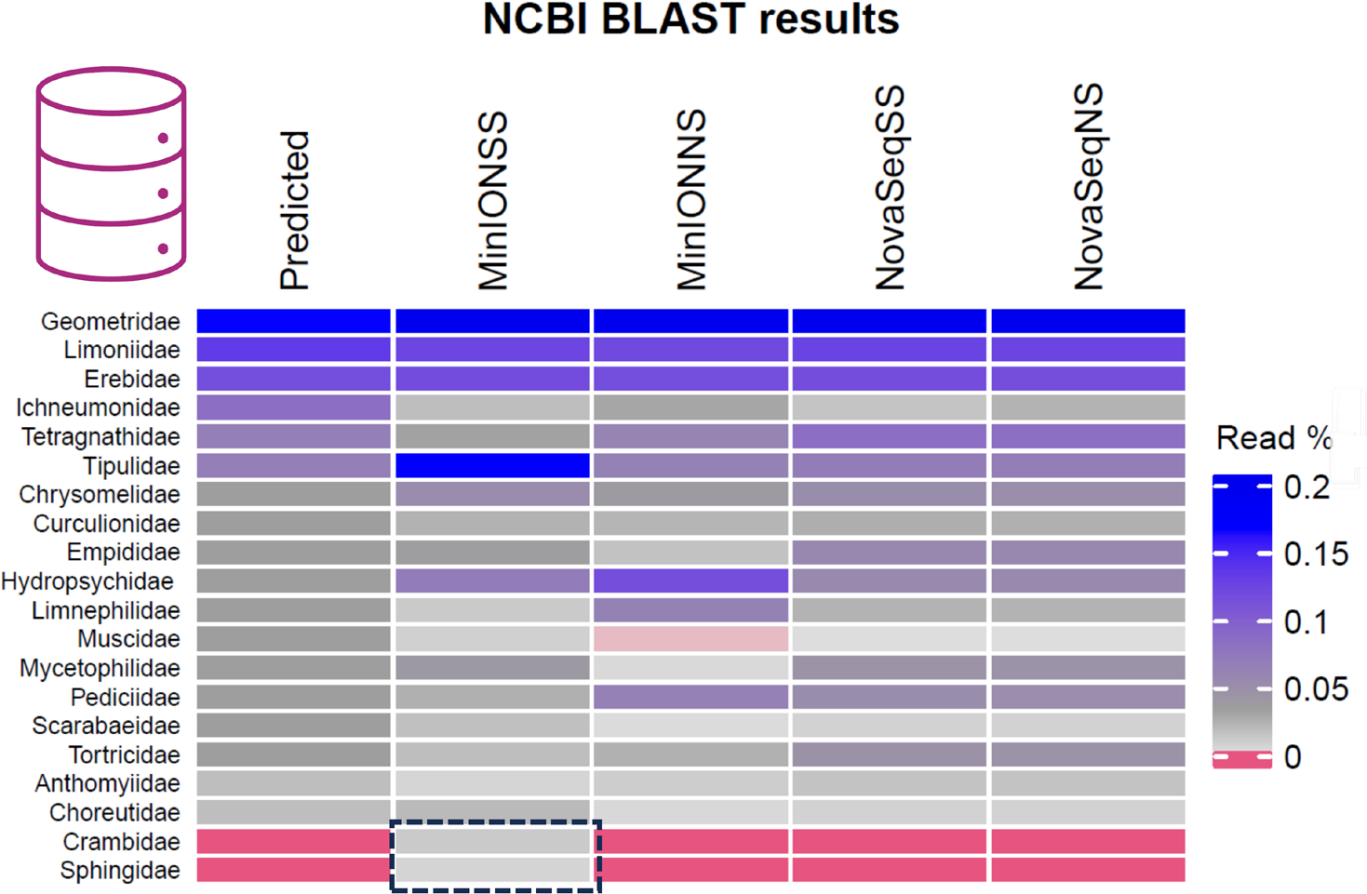
*Illumina NovaSeq* and ONT MinION family‐level taxonomic assignment using the optimum MOTU sets as determined in 3.2 against the *local Arthropod COI* (SS) and *NCBI GenBank* (NS) reference databases. Outlined in black are the false positive families.

Next, we used the MOTUs constructed by the optimum pipeline (i.e., unsupervised 97% cluster level, minimum 15 identical reads per sequence for *NovaSeq* and unsupervised 80% clustering threshold Q8, minimum 10 reads per consensus for *MinION*) as determined above to assess family‐level taxonomic assignment produced when non‐specific “NS database” (i.e., entire *NCBI GenBank Nucleotide database*) and semi‐specific “SS database” (*downloaded arthropod COI and mitogenome sequences only*) were used. Due to the high number of *MinION* MOTUs (*n* = 7633), for the NS pipeline the query file was split into eight parts, and a remote *NCBI GenBank BLAST* command was looped, allowing 1 h sleep time in order not to overload the *GenBank* server, which took approximately 2 days to complete. BLASTing the same query file against the local database consisting of only arthropod COI and mitochondrial sequences for the SS pipeline took ~30 min to complete. In terms of presence/absence, both sequencing platforms were able to detect all 18 families (i.e., no false negatives) producing strongly matched compositions to the expected one, ranging from Rho 0.82 (*p* < 0.001) for *ONT MinION* SS pipeline to 0.88 (*p* < 0.001) of *Illumina NovaSeq* NS pipeline (Figure 6).

ONT MinION SS produced the highest number of false positive families (*n* = 3), whereas for MinION NS and Illumina NovaSeq data, these taxa were removed after filtering results based on a rule that determined that a taxon was considered true if represented by at least as many or more reads than were present for the lowest true input family. Interestingly, one family—Psychodidae—was falsely present in the assemblages of all pipelines and filtering at the levels comparable to true input species and represented by several different MOTUs. This was most likely due to biological contamination from the malaise trap ethanol carryover, where many individual Psychodidae species were present together with the arthropods chosen for the AML.

The false positive family assignment resulted from MOTUs constructed from the reads with MinION sequencing errors, which were predominantly homopolymer run indels and miscalls and single G indels as well as incomplete adapter trimming (Figure S5).

### 3.3 *ONT MinION* Case Study (CS) Results

#### 3.3.1 CS Sequencing Results

A total of 11,062,289 sequence reads were generated from the ONT MinION multiplex sequencing run, resulting in a total of 293,997 reads for the five samples selected for this case study after demultiplexing (Table 2). The number of sequences mapped using *NanoPipe* varied between the sites (Table 2). A total of 89 MOTUs were generated for all sites. After filtering and removing contaminant species, a total of 46 MOTUs remained. These MOTUs represented a total of 24 species of arthropods from seven orders, as well as the host bat species, which was identified as a brown long‐eared bat ( *Plecotus auritus* ) at all five roosts.

**TABLE 2:** Total sequences for the five sites used in this study were generated by the ONT MinION sequencer using the *NanoPipe* reference clustering pipeline and the locally downloaded arthropod and mammal specific COI reference database.

Site code	Location	Total sequences	Mapped sequences	MOTUs
BB90	Tipperary	95,207	47,150	12
BB99	Offaly	54,165	38,721	21
BB101	Kildare	17,728	8854	20
BB102	Cork	64,697	59,594	12
BB104	Leitrim	62,200	29,431	24

#### 3.3.2 CS Species Richness

**FIGURE 7:**
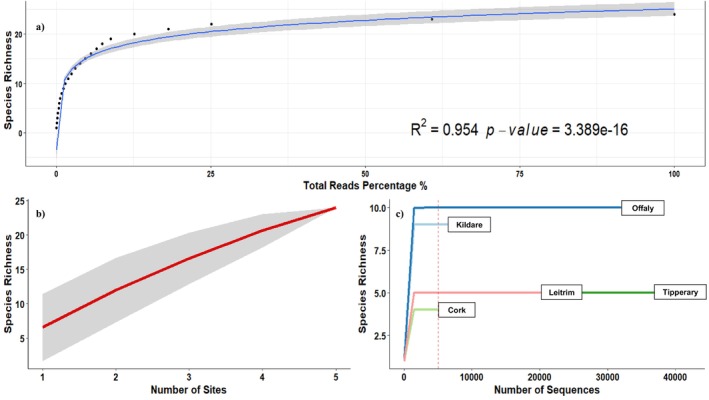
Species richness graphs and statistics for all sites (*n* = 5) using pooled faeces (*n* = 5) from *P. auritus* . (A) Cumulative total read percentage against species richness with significant relationship. (B) Species accumulation curve with standard deviation for each cumulative sampling site. (C) Rarefaction curve of sequencing depth for total species richness retrieval. Estimated sequencing depth for retrieval of all observed arthropod species indicated by dashed red line (5020 reads).

Species richness from all five sites sampled peaked at a maximum of 24 species at the Leitrim roost (Figure 7a,b). However, by sequencing DNA from only five bat droppings, we likely under‐sampled the arthropod diversity in these roosts as the extrapolated species richness was estimated at 43 ± 13 SD (Chao et al. 2009) and the species accumulation curve did not reach the asymptote (Figure 7b). As little as ~25% of sequencing coverage of total reads was necessary to return the majority (~85%; 22 of the 24) arthropod diet species (Figure 7a). Thus, species richness was shown not to be significantly affected by the total read depth, with most of the observed species being identified with relatively low read coverage (*p* < 0.001, *R* ^2^ = 0.954). This was confirmed using rarefaction curves for each site, which estimated a total read amount of 5020 as a sufficient sampling depth to retrieve all observed arthropod species (Figure 7c).

#### 3.3.3 CS Diet Composition

**FIGURE 8:**
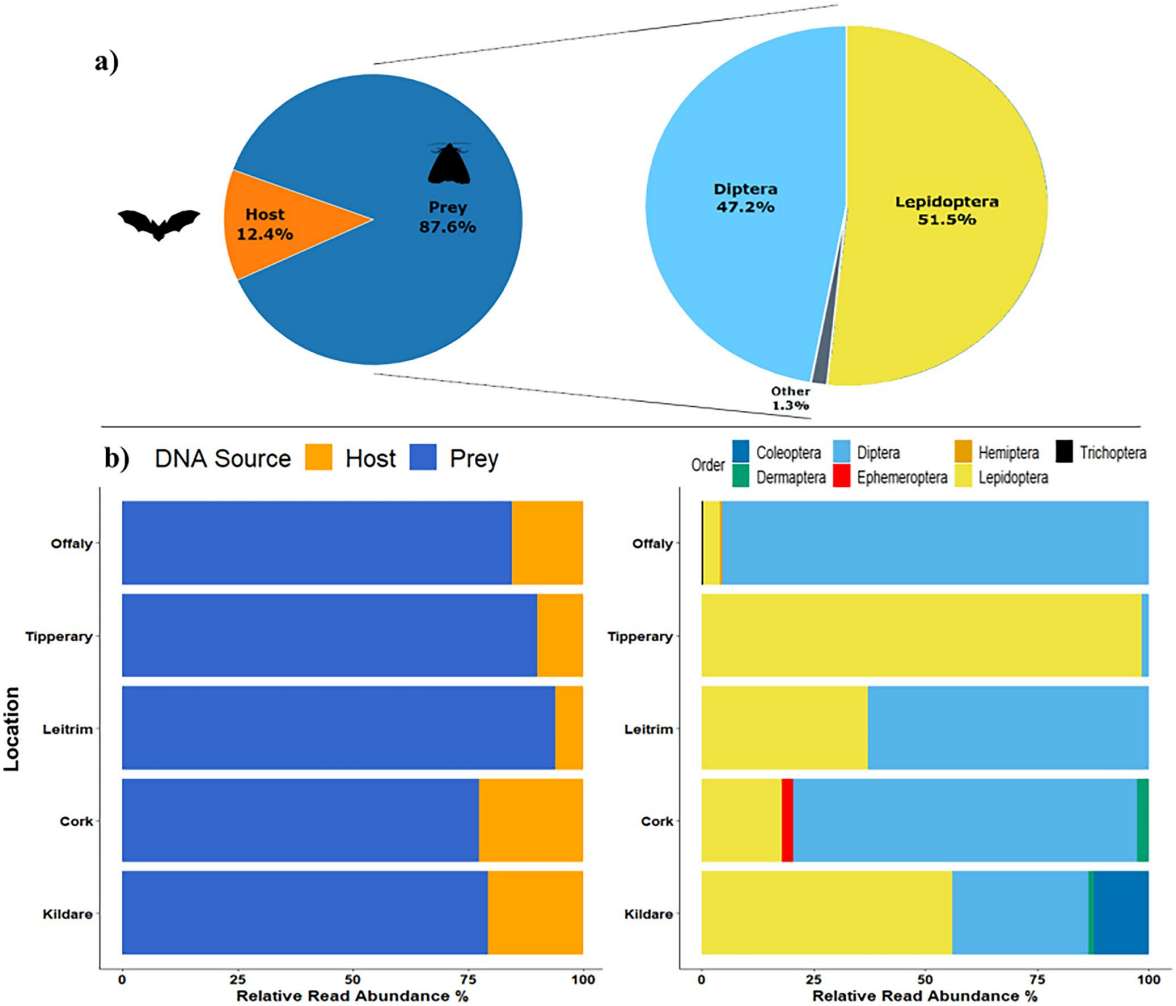
Relative read abundance (RRA) of taxonomic assignment for five sites of *P. auritus* . (A) Pie chart showing RRA of host DNA against prey DNA, with a larger pie chart showing RRA of the prey DNA as breakdown between arthropod orders. (B) Stacked bar plots showing RRA for each individual site for both host DNA and prey DNA, as well as the breakdown of the arthropod orders RRA.

Out of the total of 122,104 reads retrieved from the *MinION* sequencing run for the five CS sites, 106,967 originated from the prey DNA and 15,137 were from the bat hosts, i.e., *P. auritus* (Figure 8a,b). Similarly, the majority of the MOTU sequences were attributed to prey DNA at each individual site using relative read abundance (RRA). In terms of arthropod prey, we retrieved 7 orders, 12 families, 21 genera, and 24 species (Table S7). The majority of the overall diet using RRA constituted of Lepidoptera (51.5%) and Diptera (47.2%), while the other five orders only accounted for 1.3% of total RRA (Figure 8a). On an individual site level, there was a difference in diet RRA, with three of the five (Offaly, Leitrim and Cork) composing mostly of dipterans and less of lepidopterans (Figure 8c). All sites contained both Lepidoptera and Diptera, while the other five orders—Coleoptera, Dermaptera, Ephemeroptera, Hemiptera, Trichoptera—appeared in only two or fewer sites. Dermaptera appeared in two sites (Cork and Kildare), while Coleoptera (Kildare), Ephemeroptera (Cork), Hemiptera (Offaly) and Trichoptera (Offlay) only appeared in one site.

## 4 Discussion

DNA metabarcoding has developed to rapidly produce millions of sequences which can identify species present in a sample (Alberdi et al. 2018). This is of particular importance when studying the diet composition of highly digested prey, such as in bats, as traditional morphological studies often have poor taxa resolution (Nolan et al. 2023). The feasibility of sequencing multiple species in parallel has proven to provide an accurate dietary composition from a small amount of eDNA even from highly processed sources with a degraded DNA (Alberdi et al. 2018; Esnaola et al. 2018; Galan et al. 2018; Jusino et al. 2019). Thus, using eDNA metabarcoding to characterize the diets of organisms that are difficult to track and monitor by field methods, such as bats, can be highly beneficial for researchers studying ecology and behavior (Taberlet et al. 2018).

ONT MinION nanopore sequencing is a relatively new third‐generation high‐throughput sequencing technique that is rapidly gaining popularity in molecular DNA metabarcoding studies. This is largely due to its small instrument size and relatively low maintenance costs, which offer the independence and flexibility to obtain real‐time or near real‐time results. However, the main drawbacks are reportedly higher sequencing error rates and less established bioinformatic analysis pipelines, compared to NGS platforms such as Illumina MiSeq, HiSeq, and NovaSeq. Since molecular DNA metabarcoding is rapidly becoming an irreplaceable tool in a wide variety of life sciences, having a portable “in‐house” sequencing device would provide access to augmented and speedy biodiversity monitoring for many researchers and stakeholders. Therefore, motivated by the relative scarcity of studies that are testing the suitability of ONT MinION for accurate biodiversity assessments from short‐read amplicon sequencing such as mitochondrial *COI*, we compared the performance of the ONT MinION relative to Illumina NovaSeq. We first a generated a mock sample with known input species barcodes that was processed by currently available and broadly used metabarcoding bioinformatic pipelines to: (1) determine the precision of the taxon retrieval; (2) possible sources of errors; (3) algorithm configurations that produce the closest results to the expected input; and (4) the effect of the reference database choice on accurate species composition retrieval. We then applied this information to test ONT MinION sequencing on a real‐life test sample from the field using the droppings collected at bat roosts in Ireland to demonstrate the feasibility and inform on the necessary considerations when implementing a successful MinION short‐read metabarcoding study.

### 4.1 AML‐Based MinION Taxon Retrieval Accuracy Assessment

Although Illumina NovaSeq outperformed the ONT MinION in terms of correct read assignment to the species that were expected from the arthropod mock sample, if unmapped reads for MinION were not considered, the discrepancy was not large, with both platforms providing a moderate correspondence (Spearman's rank Rho = 0.62, *p* < 0.001 vs. Rho = 0.57, *p* = 0.001 for NovaSeq and MinION, respectively). Both instruments successfully detected all 30 input species (i.e., no false negatives), although *P. errans* and *S. brunnea* were highly misrepresented with only ~3% and 18% of the predicted output reads assigned for *P. errans* and *S. brunnea* , respectively for both sequencing platforms. Given that both species did not have sufficient read representation despite having the exact reference sequence in datasets produced by both sequencing platforms, we conclude that this is most likely due to an amplicon pooling error, primer bias or inaccurate *Qubit* concentration measurement rather than a shortcoming in sequencing workflows. We acknowledge that these issues could have been prevented by including several technical replicates, which we lacked due to available labour and sequencing costs. Similarly, we considered the overrepresentation of the two species— *H. pellucidula* and *L. marginata* —in both sequencing platform datasets may be a result from deviation of equimolar concentrations or primer bias in the AML rather than metabarcoding workflow flaws. Removal of these species improved the composition closeness to the predicted input for both platforms (Spearman's rank Rho = 0.70, *p* < 0.001 and Rho = 0.66, *p* < 0.001 for NovaSeq and MinION, respectively [Table S6]). This showed that, if a highly accurate reference sequence database is used to guide MinION MOTU construction, the MinION results are highly comparable to Illumina.

On family‐level assignment, Illumina NovaSeq provided strong correlation to the predicted taxon composition even when non‐specific (total *NCBI GenBank*) and semi‐specific (arthropod *COI*) databases were used as a reference, whereas the ONT MinION was somewhat less efficient (Spearman's rank Rho = 0.82, *p* < 0.001 vs. Rho = 0.79, *p* < 0.001 for NovaSeq and MinION, respectively). There were no false negatives for MinION, but when a semi‐specific database was used for taxon assignment MinION MOTUs obtained via unsupervised clustering were assigned to two false positive families—Crambidae and Sphingidae, whereas NovaSeq did not detect any false positives after the removal of families that were represented by less than 0.03% of the total reads. We speculate that this is due to an inaccurate alignment resulting from MOTU's constructed from reads with sequencing errors, particularly if intraspecies diversity is high.

### 4.2 *MinION* Filtering Parameter Effect

As a result of the errors occurring during the amplification or sequencing steps, or incomplete adapter trimming, the number of unique reads representing each individual is usually higher than the number of individual biological specimens actually present in the sample (Bonin et al. 2023). Further, depending on the diversity of the selected marker region and true intraspecific diversity, these errors may also lead to an over‐represented number of taxa (i.e., false positives). Sequence clustering is one of the approaches used to reduce these biases by collapsing the sequences above a certain similarity threshold into one single MOTU and keeping the most abundant sequence of the cluster as an individual taxonomic entity representative. Thus, choosing the correct clustering threshold is critical as the threshold that is too low will collapse different taxa into the same MOTU (over‐merging), while a threshold that is too high can create too many MOTUs (over‐splitting) compared to the actual level of diversity (Bonin et al. 2023). However, the choice of the clustering threshold is rarely justified. Because of the different technologies behind the two sequencing platforms and a very different profile of the reads (i.e., short vs. long‐read sequencing), separate filtering strategies and pipelines were applied for ONT MinION and Illumina NovaSeq sequencing. The most accurate clustering method for ONT MinION was using a reference‐guided approach via the *NanoPipe* webtool rather than unsupervised *de novo* clustering (i.e., VSEARCH). *NanoPipe* is a fast and intuitive platform, and it correctly created all of the 30 consensus MOTUs representing each of the input species, with the majority of the sequence reads (92.3%) mapped to these MOTUs.

In terms of read quality, the highest accuracy was retrieved with the Q8 read dataset, i.e., more reads with lesser Phred scores were better. The same was true for the cluster depth (reads per cluster centre) as datasets with 10 and 50 reads rather than 100 reads per cluster slightly improved retrieved species composition for MinION. Although ONT MinION quality scores are also encoded with ASCII characters from 33 to 126 (the higher the value, the better the quality expected), representing Phred scores 0–93, MinION quality scores do not follow Phred expected error rates (Delahaye and Nicolas 2021; Laver et al. 2015). However, the recorded ONT quality scores (Q) are correlated with the sequencing error rates (E) and a minimum threshold of Q7 is arbitrarily recommended for acceptable ONT data quality (Delahaye and Nicolas 2021). Our results were in agreement with this threshold and, in fact, indicated that over‐filtering the raw reads may negatively affect MinION results.

### 4.3 Effect of the Reference Database and Taxonomic Assignment

Our mock dataset suggests that out of all the considerations, it is the incompleteness of barcode reference sequences that is likely to have the most critical impact on successful taxonomic retrieval, and this effect was greater for the MinION than for Illumina data, especially in terms of producing falsely positive taxa. Given that significant taxon representation gaps still exist in DNA databases, e.g., mislabelling, sequencing errors, low taxonomic resolution, missing taxa, missing intraspecific representation, etc. (Dopheide et al. 2019; Keck et al. 2023), which therefore may limit the current operational capacity of MinION metabarcoding. However, our mock sample analysis echoes the conclusion by Srivathsan et al. (2022) demonstrating that MinION is indeed already suitable for the analysis of metabarcoding data, provided that the species diversity per sample is relatively small and that the target species have a good representation in publicly available databases and is also highly geographic region specific (Dopheide et al. 2019). With ever‐decreasing sequencing costs and initiatives such as the *Earth Biogenome Project*, which aims to sequence the genomes of all eukaryotic life on earth (Consortium 2022), the future in terms of representation of the taxa looks promising. Therefore, the biggest immediate constraints that need to be addressed by the metabarcoding community are the establishment of universal reference databases within the sequencing data processing pipelines that are user‐friendly and readily transferable format‐wise, which would save time taken for sample processing.

### 4.4 Impact of *MinION* Sequencing Errors

The main known limitation of the ONT sequencing technology is its ability to call the correct number of bases in homopolymer runs (Rang et al. 2018; Whitford et al. 2022; Wick et al. 2019). Having analysed the MOTUs that represented the false positive families by comparing them to the corresponding Sanger reference sequence, we found that the discordance indeed originated from MinION sequencing artefacts because of falsely called homopolymer base numbers, as well as single guanine indels. The second source of error was single nucleotide variants between reference sequence and MOTU sequence at the 5′ and 3′ ends of the sequences, which we assigned to incomplete nanopore adapter trimming by *Porechop*. In terms of accuracy, the newly released successor R10.4.1 ONT flow cells paired with the Kit 14 chemistry, which are compatible with MinION and GridION devices, are new promising options as they are designed to deliver higher manufacturer's reported consensus accuracy of above 99% owing to increased depth by sequencing both strands of double‐stranded DNA molecules, optimised algorithms, and improved nanopore structure. This is expected to improve the first two issues. Shortly after the sequencing was performed for this study (March 2022), ONT developed the ‘short fragment mode’ (ONT 2022), allowing reads as short as 20 nt to be included. This should increase the consensus sequence accuracy by also retaining short reads (< 200 nt) that previously were specifically eliminated from the datasets by basecalling software to avoid adapter‐only reads and may have prevented the latter issue. Our findings demonstrated that even the 9.4.1 flow cell could yield sufficiently reliable results for metabarcoding applications thus establishing a valuable benchmark. The enhanced accuracy of 10.4.1 flow cells and further iterations will offer clear advantages for future research.

### 4.5 Sequencing Affordability and Practical Recommendations

ONT MinION is often referred to as a low‐cost sequencing alternative to traditional Illumina high‐throughput platforms suitable for smaller research groups (Van Der Reis et al. 2023; Santos et al. 2020; Mikheyev and Tin 2014). However, with the sequencing market exhibiting solid growth in recent years and an increasing number of companies offering to outsource next generation sequencing at cost‐effective rates, the costs of traditional NGS services are continuously decreasing. Furthermore, the “hidden costs” such as sample multiplexing barcodes, third‐party reagents, and high flow‐cell costs are rarely considered and can significantly increase the library preparation costs (to several thousand EUR), especially for one‐off projects for which the reagents will not be re‐used or shared between laboratories. Another consideration we highlight in this study is the coordination of sample collection, DNA extraction, and library preparation with the delivery of ONT flow cells (~1‐week delivery time) as the shelf life for MinION flow cells is two–three months to guarantee optimum sequencing output (Figure S6). The maximum output of 50 Gb for each flow cell should also be considered.

### 4.6 Applied Metabarcoding of Mini‐Barcode Regions Using ONT MinION

To test the feasibility of using ONT MinION for metabarcoding of real‐life samples, we used Irish bat faecal DNA to sequence the mini‐barcode regions designed for host and arthropod diversity characterisation. This is to our knowledge the first time that metabarcoding has been performed using nanopore technology in any bat diet characterisation study published. The host in all five roosts was genetically identified as Brown long‐eared bat ( *P. auritus* ), which is one of the most commonly observed species of bats in Ireland (Harrington et al. 2019). Our MinION metabarcoding results showed that the *P. auritus* diets in September mainly consisted of Lepidoptera and Diptera, with the other five orders accounting only for 1.3%. This was in close agreement with the studies that investigated the droppings of Irish Brown long‐eared bats using morphological methods. The most comprehensive examination by Shiel et al. (1991) also showed that the total May–October *P. auritus* diet in Western Ireland consisted of nearly two‐thirds of Diptera and Lepidoptera (in roughly equal proportions). The rest of the diet was comprised of Trichoptera, Arachnida, Chilopoda, Coleoptera, Dermaptera, Hymenoptera, and Hemiptera, out of which four were also detected by our MinION metabarcoding. Ephemeroptera was not recorded by Shiel et al. (1991) but was only present in one of our roosts in Co. Cork and has been known to be highly sensitive to water pollution (Beketov 2004); therefore, this may be because of high small‐scale regional variation. The *P. auritus* diet presented here is also supported by other metabarcoding studies in Europe with recovery of predominantly lepidopteran and dipteran species in similar proportions to what we show here (Andriollo et al. 2019; Andriollo et al. 2021; Starik et al. 2021). Further, *P. auritus* is known as both a foliage gleaning and an aerial hawking predator (Entwistle et al. 1996). Our MinION results confirmed this and showed that although the Irish Brown long‐eared bats mostly preferred flying insects, they were able to switch their foraging strategies to also feed on non‐flying arthropods such as earwigs (i.e., *Forficula auricularia* ), which further supports the reliability of the results.

Based on extrapolated species richness and comparison to other studies (Andriollo et al. 2021; Shiel et al. 1991) we conclude that our study most likely greatly underestimated the total diversity of the Irish *P. auritus* diet. This was mostly due to insufficient sampling in terms of the number of droppings per roost and the number of roosts rather than the sequencing strategy employed. To fully capture the diet diversity for *P. auritus* , we suggest increasing the number of sites and also the number of droppings from each roost. As an indication, Andriollo et al. (2019) estimated that 15–20 pellets taken over a two‐week period likely represented the cumulated diet of the entire *P. auritus* maternity roost. Further, we advise using primer combinations with one set to amplify both host and prey (e.g., Vamos) and another set for solely prey DNA amplification (e.g., Zeale). With this strategy, we achieved the desired balance of being able to detect bat host (i.e., *P. auritus* ) while having the majority of reads assigned to arthropods (87.6%), which maximised computational resources and sequencing costs. The pooling of faeces was necessary to reduce the cost of the experiment, as individually sampled faeces would require barcode multiplexing and thus reduce the number of sites sequenced on the flow cell and Andriollo et al. (2019) also showed that pooling samples uncovered greater dietary diversity than sequencing individual *P. auritus* droppings. Of note, our results showed that MinION sequencing depth had less of an effect on species richness than sampling effort. Most species were recovered by a relatively low total read abundance of ~20% of total reads (Figure 7a) and required only 5020 reads per site to recover the 24 arthropod species. This further confirms that the number of biological replicates was the main contributing factor in this study to accurately describe *P. auritus* diet. This is useful for future metabarcoding studies, as it shows that ONT nanopore's relatively low data output capacity compared to NGS options does not pose a constraint.

We also found that once the basecalling and demultiplexing was performed, the simplest and fully automated “click and drag” pipeline for further filtering, clustering and MOTU construction using the *NanoPipe* webserver was in fact the most suitable approach and only limited to the accuracy and comprehensiveness of the reference sequences. This may be an issue to some researchers as retrieving an accurate, comprehensive and target taxa‐specific database for their particular study can be problematic (Keck et al. 2023). This is important as the current bioinformatic pipelines used in the metabarcoding field require a relatively high level of expertise and are not necessarily applicable to all researchers or ecologists, such as ecological consultants.

### 4.7 Future Directions

There is a growing need for easy‐to‐use filtering and clustering pipelines and open online barcode databases that are regularly maintained and updated. Our results show that existing clustering tools designed for nanopore long‐read whole genome assemblies such as *NanoPipe* hold a great potential and could be further adapted for complete short‐read metabarcoding analysis, especially if mini‐barcode reference databases were also made available on the same platform. Up until very recently, the main barriers for nanopore application in short‐read amplicon metabarcoding were the sequencing error and basecalling algorithms designed for long reads, as well as a lack of reference sequences. With continuous technology development, the error rate of nanopore sequencing raw reads is approaching 1% (Q20); the biggest remaining challenge is accurate taxon assignment. Further, the standardisation and transferability of existing reference databases and dataset tools and formats such as COins (currently only in QIIME format) (Magoga et al. 2022), *ecoPCR* (Boyer et al. 2016) (custom format part of OBITOOLS) and *GenBank* (needs to be converted to BLAST database) would greatly contribute to our effectiveness in promoting the quality of the research efforts.

## 5 Conclusions

Our preliminary research has demonstrated that it is feasible to use nanopore for short‐read metabarcoding studies to retrieve comparable results to Illumina platforms and provide reliable real‐life species compositions without sophisticated bioinformatics efforts using a click‐and‐drag web analysis tool. Further refinements are required to reduce nanopore sequencing costs and improve reference database accessibility, which will greatly facilitate biodiversity monitoring and informed real‐time on‐site decisions. We hope that the information provided here will aid in planning and budgeting nanopore metabarcoding studies to maximise biodiversity detection.

## Author Contributions

**James M. Nolan:** conceptualization (lead), data curation (lead), formal analysis (lead), formal analysis (lead), investigation (lead), investigation (lead), methodology (lead), methodology (lead), project administration (equal), project administration (equal), visualization (lead), visualization (lead), writing – original draft (lead), writing – original draft (lead), writing – review and editing (lead), writing – review and editing (lead). **Ilze Skujina:** conceptualization (lead), data curation (lead), formal analysis (lead), investigation (lead), methodology (lead), project administration (lead), validation (lead), visualization (lead), writing – original draft (lead), writing – review and editing (lead). **Gwenaëlle Hurpy:** funding acquisition (equal), investigation (supporting), methodology (supporting), project administration (supporting), resources (equal), writing – review and editing (supporting). **Andrew J. Tighe:** resources (supporting), software (supporting), writing – review and editing (supporting). **Conor Whelan:** methodology (supporting), project administration (supporting), writing – review and editing (supporting). **Emma C. Teeling:** conceptualization (equal), data curation (equal), formal analysis (equal), funding acquisition (lead), investigation (equal), methodology (equal), project administration (equal), resources (lead), software (lead), supervision (lead), validation (equal), visualization (equal), writing – original draft (equal), writing – review and editing (equal).

## Conflicts of Interest

The authors declare no conflicts of interest.

## Supporting information


Figures S1–S6.

Tables S1–S7.

## Data Availability

Raw sequencing data have been made available on NCBI SRA, data set (PRJNA1152142). Metadata, raw sequencing reads, intermediary results, main analysis scripts, and supporting figures are deposited in Dryad with DOI http://datadryad.org/stash/share/SHzymX2lTaWXHc04pIZdlqClZRDB6Jah49Apbjif9Pk (Temporary URL). New COI barcode sequences have been deposited in the *GenBank NCBI* nucleotide database (accession nos. PP759737‐PP75766: PQ187542‐ PQ187543).
