# Supplementary material for: Evaluation of Oxford Nanopore Technologies MinION Sequencer as a Novel Short Amplicon Metabarcoding Tool Using Arthropod Mock Sample and Irish Bat Diet Characterisation
Source: Ecol Evol. 2025 May 4;15(5):e71333. doi: 10.1002/ece3.71333 (PMC12050260; doi:10.1002/ece3.71333)
Supplement: Supplementary file 1 — Figures S1–S6. [file ECE3-15-e71333-s002.pdf]

Supplemental Information for:

Evaluation of *Oxford Nanopore Technologies MinION* sequencer as a novel short amplicon metabarcoding tool using arthropod mock sample and Irish bat diet characterisation.

James M, Nolan, Ilze Skujina, Gwenaëlle Hurpy, Andrew J. Tighe, Conor Whelan, Emma C. Teeling.

Table of Contents:

**Figure S1**	Page 2
**Figure S2**	Page 3
**Figure S3**	Page 3
**Figure S4**	Page 4
**Figure S5**	Page 5
**Figure S6**	Page 6

Supplementary Figure 1. A box-and-whisker plot showing aggregated Phred quality score statistics at each position along all reads length-filtered AML *ONT MinION* reads. The red line within each yellow box represents the median quality score at that position/window. Yellow box is the inner-quartile range for 25th to 75th percentile. The upper and lower whiskers represent the 10th and 90th percentile scores. The background of the graph divides the y axis into very good quality calls (green), calls of reasonable quality (orange), and calls of poor quality (red) according to the conventional *Illumina* cut-off values.

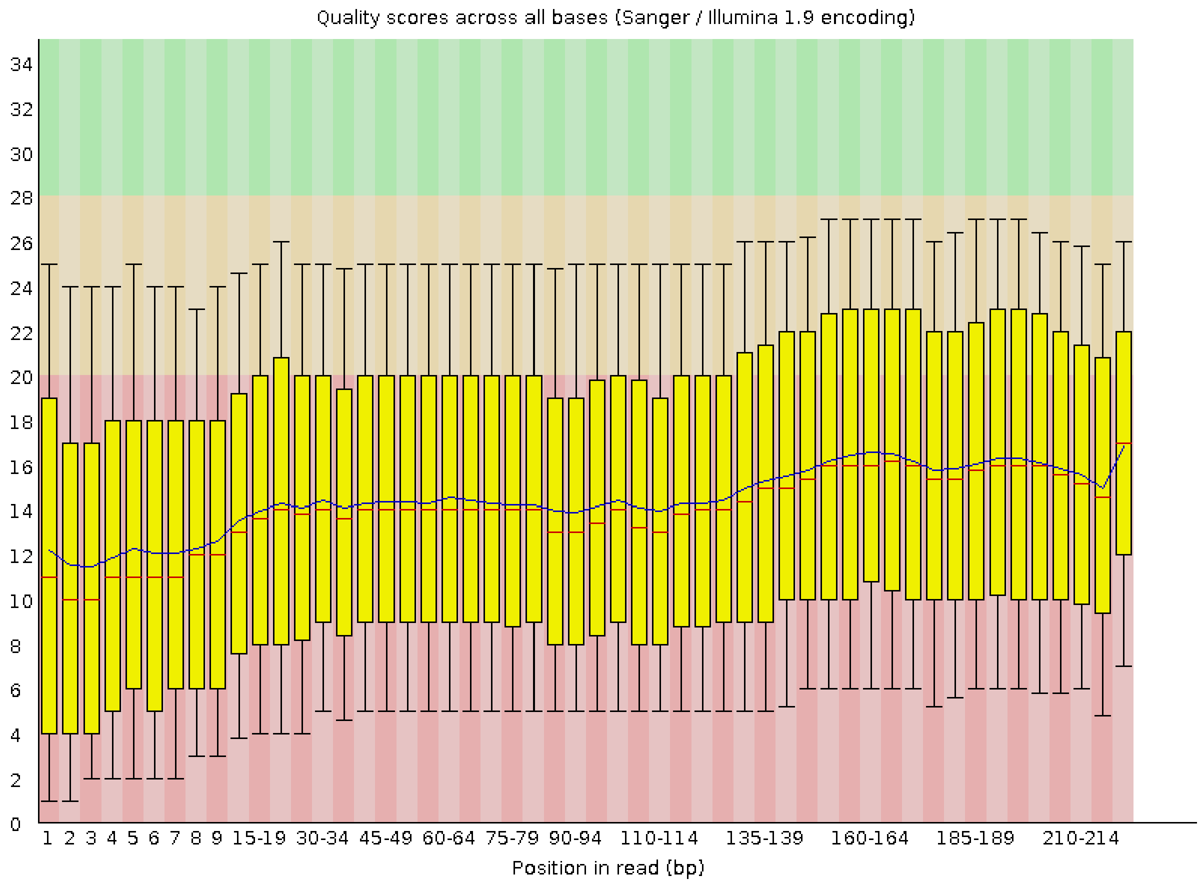

Supplementary Figure 2. Weighted Histogram of raw AML *ONT MinION* read lengths after log transformation.

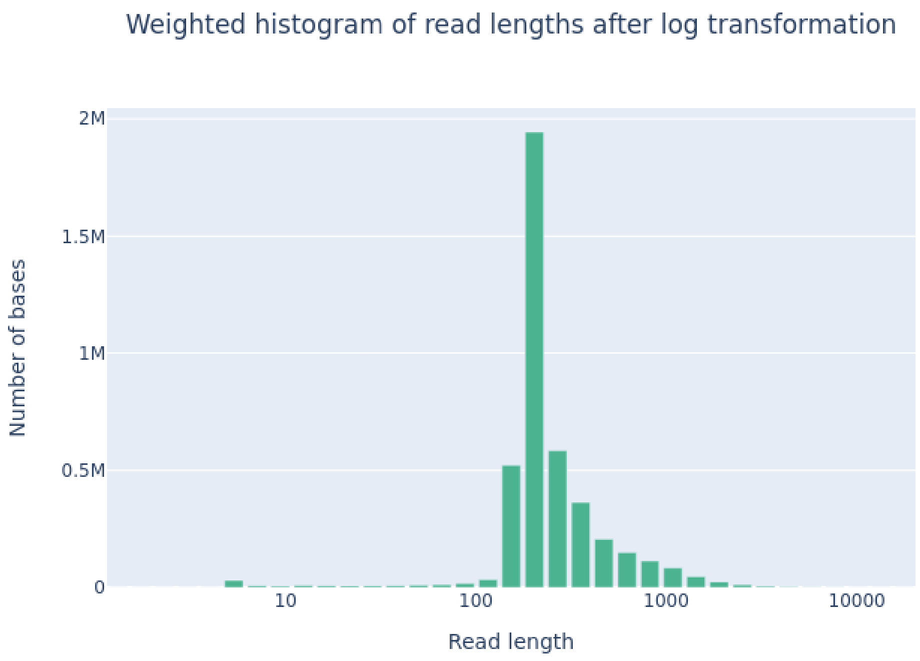

Supplementary Figure 3. Raw AML *ONT MinION* read yield by length.

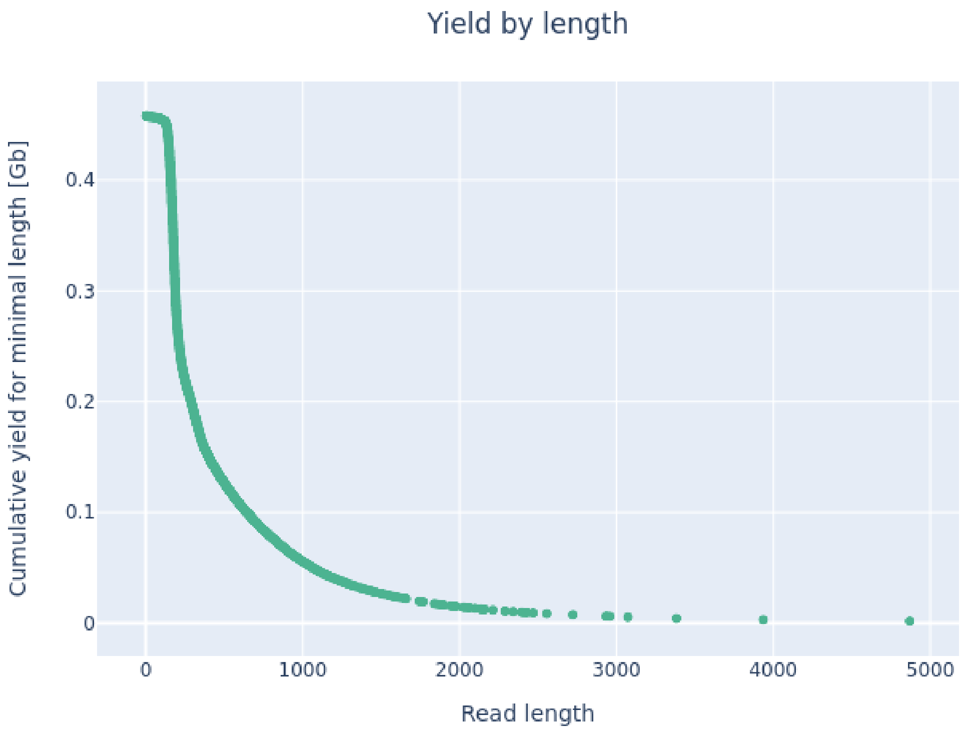

Supplementary Figure 4. A box-and-whisker plot showing aggregated Phred quality score statistics at each position along all reads in the file for merged AML *Illumina NovaSeq* reads. The red line within each yellow box represents the median quality score at that position/window. Yellow box is the inner-quartile range for 25th to 75th percentile. The upper and lower whiskers represent the 10th and 90th percentile scores. The background of the graph divides the y axis into very good quality calls (green), calls of reasonable quality (orange), and calls of poor quality (red) according to the conventional Illumina cut-off values.

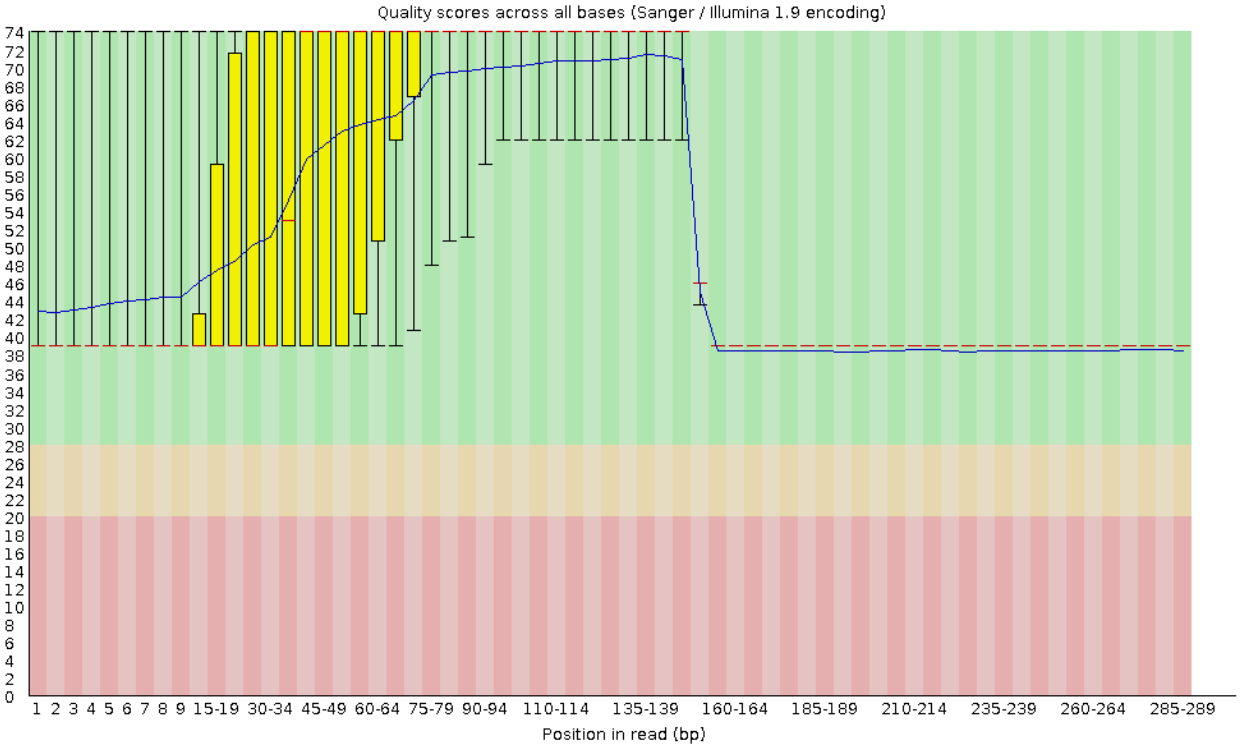

Supplementary Figure 5. *MinION* false positive family MOTUs (MOTU_000734525 (19 reads), MOTU_000351835 (75 reads) and MOTU_000121140 (231 reads)) when using SS (local arthropod COI database) assignment aligned to the respective HS, SS and NS closest reference sequences. Highlighted in yellow is a SNP specific for each haplotype positioned outside the minibarcode regions. Highlighted yellow are *MinION* sequencing errors.

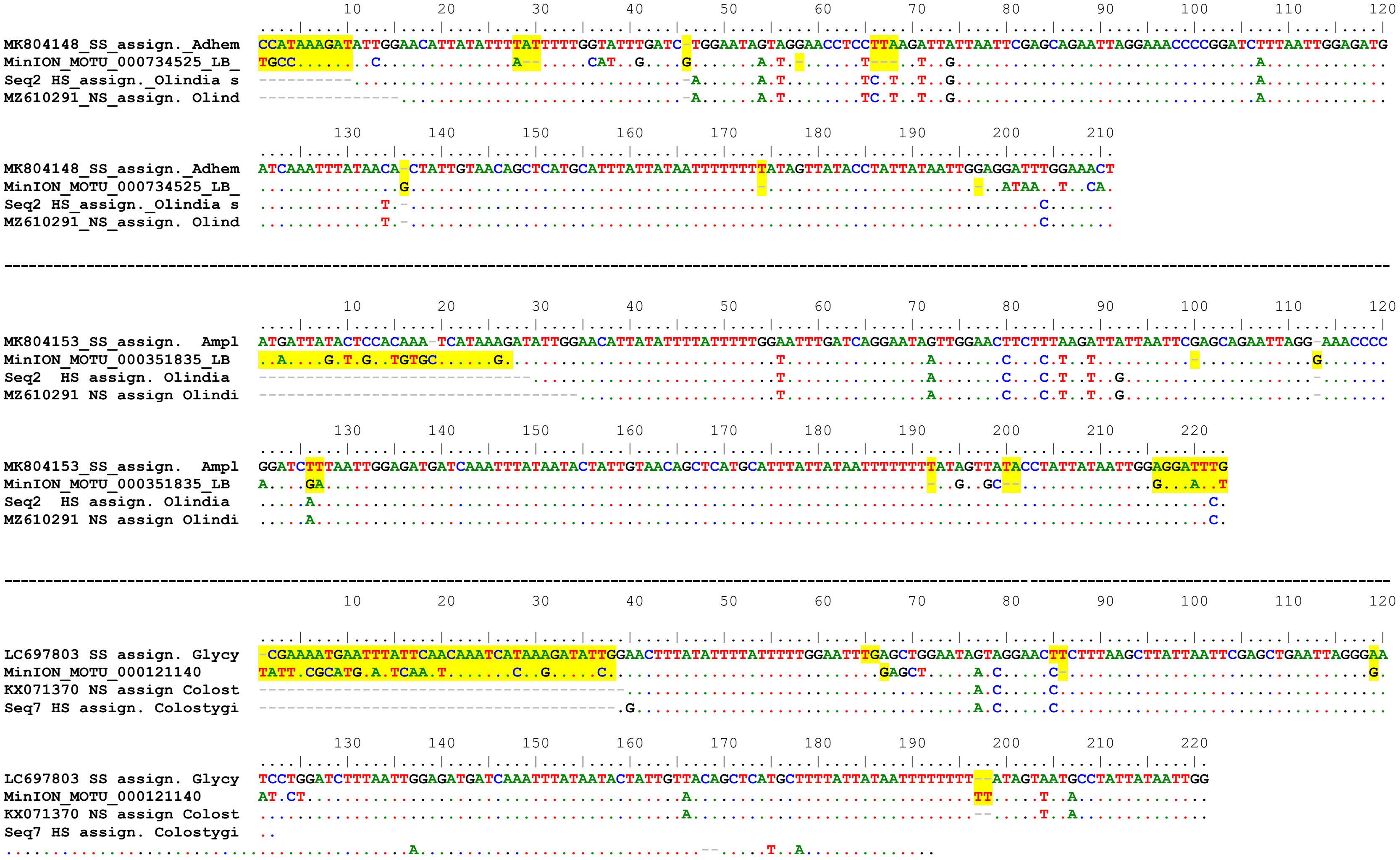

Supplementary Figure 6. *Cost efficiency and practical considerations*

It should be noted that the total per sample costs are based on economy-of-scale Illumina sequencing by outsourcing a commercial service provider (e.g. *Novogene*), whereas *ONT* metabarcoding libraries were prepared and sequenced in-house and thus required to purchase third party modules (i.e. *NEBNext*) and barcode sets, which are reusable. However, we found that even when basing the costs of maximum number of samples that can be sequenced per each reagent kit and third party reagents, ONT MinION in-house sequencing was considerably more expensive (nearly 10x) than Illumina NovaSeq in this particular setup (i.e. EUR 2.92 vs EUR 0.30 per 1 Mb of data, respectively see Sup. Table 9). Moreover, providing that the sequencer and computer is already available, the minimum initial investment of at least EUR 2,400, which included the flow cell, sequencing reagent kit and third party end-repair kit was needed to perform any of the MinION sequencing, which could pose an obstacle for smaller less well-funded groups.

Another consideration is the ONT flow cell expiration times with optimum recommended storage of several months which may prevent cheaper option of bulk-buying and does not guarantee the number of available sequencing pores as wells as requires careful planning and readiness of the samples. In contrast, shipping the samples to a sequencing provider was possible at any time and ensured guaranteed minimum amount of data for libraries that satisfy the required quality control.

	Via Service Provider	*In House*	
	NovaSeq	***MinION*** (One-off use)	***MinION*** (Max-use per sample*)
Data Output: Mb	950	700	7.29
Data Output: No. of reads	7,338,142	1,910,000	19895.83
Shipping costs (EUR)	145	--	--
Sequencing service (EUR)	144	--	--
R9.4.1 Flow cell (EUR)	--	810	8.44
SQK-LSK109 Ligation Seq. kit (EUR)**	--	575	1.00
E7546L End Repair (EUR)**	--	1,045.13	10.89
EXP-NBD196 Native Barcoding (EUR)**	--	--	0.94
**Total (EUR)**	**289**	**2430.13**	**21.26**
**Price per 1 Mb data (EUR)**	**0.30**	**3.47**	**2.92**
**Price per 1000 raw reads (EUR)**	**0.04**	**1.27**	**1.07**

**Costs calculated if maximum of samples are used per reagent kit.*

***Cheaper alternative available, but sequencing output may be reduced.*
